# A network analysis of lifetime stressor exposure, mental health, well-being, and immune cell mobilisation to acute stressors in young adults

**DOI:** 10.1016/j.bbih.2026.101186

**Published:** 2026-01-26

**Authors:** Ella McLoughlin, Daniele Magistro, Roberto Vagnetti, George M. Slavich, James E. Turner, Rachel Arnold, Lee J. Moore, John Hough

**Affiliations:** a School of Science and Technology, Nottingham Trent University, UK; b School of Health Science, Faculty of Environmental & Life Sciences, University of Southampton, UK; c School of Environment, Education, and Development, University of Manchester, UK; d Department of Psychiatry and Biobehavioral Sciences, University of California, Los Angeles, USA; e School of Sport, Exercise, and Rehabilitation Sciences, University of Birmingham, UK; f Department for Health, University of Bath, UK

**Keywords:** Adversity, Early life stress, Health, Immunity, Lymphocytes

## Abstract

Many young adults experience mental ill-health which is increasing over time. From a theoretical perspective, the accumulation of stressors experienced over the lifespan may be an important factor in influencing the mental health and well-being of young adults. Although continued exposure to stressors can negatively impact aspects of immunity, researchers have yet to examine how lifetime stressor exposure (i.e., frequency and severity) influences mental ill-health and well-being, and how these states subsequently affected immune cell mobilisation in response to a laboratory-based social stressor in young adults. Eighty-six participants (*M*_*age*_ = 23.31 years, *SD* = 4.94) completed an online questionnaire which assessed their exposure to lifetime stressors, symptoms of depression and anxiety, and levels of well-being. Next, participants completed the Trier Social Stress Test while immunological (i.e., lymphocytes, monocytes and neutrophils) data were collected immediately pre and post the test. Results revealed that the more frequent and severe stressors experienced during early life rendered individuals more susceptible to stressors during adulthood, which positively influenced symptoms of depression and subsequent anxiety. These aspects then deterred well-being, which negatively affected immune cell mobilisation to the acute stressor. The results highlight the potential importance of assessing lifetime stressor exposure for researchers and clinicians aiming to study the social-environmental drivers of poor immune and clinical health.

## 1 Introduction

Given the recent increase in depression rates in youth, with 44 % of high school students experiencing persistent feelings of sadness or hopelessness (Centers for Disease Control and Prevention, 2023), the importance of optimal health and well-being in young adults has received growing attention (Wyn, 2022). A key aspect of optimal health is mental health, which the World Health Organisation (WHO) has defined as “a state of well-being in which the individual realises his or her own abilities, can cope with the normal stresses of life, can work productively and fruitfully, and is able to make a contribution to his or her community” (WHO, 2004, p. 12). Based on Keyes' (2002) conceptualization, mental health and mental illness are distinct yet interconnected dimensions, with mental illness reflecting the presence or absence of psychological disorders (e.g., depression) on one continuum, and mental health representing a complete state, ranging from distress to flourishing, on a separate but related continuum. Evidence suggests that a substantial number of young adults experience mental ill-health, with statistics highlighting that this has worsened over time. Indeed, the National Health Service (NHS) estimated that 700 000 young adults are in contact with the health service for mental ill-health. In the same report, it was also highlighted that 23 % of 17 to 19-year-olds and 22 % of 20 to 25-year-olds had a probable mental condition (NHS England, 2023). Beyond the United Kingdom, ∼25 % of young adults in Australia experiencing psychological distress, a ∼20 % rise since 2012 (Mission Australia, 2019). The rate of young adults reporting major depression in the United States increased by ∼65 % between 2008 and 2017 (Twenge et al., 2019). Understanding and addressing mental ill-health during young adulthood is of vital importance, as ∼50 % of mental conditions first onset in mid-adolescence and ∼75 % start in mid-twenties (Kessler et al., 2005).

According to the integrative model of lifespan stress and health, the cumulative effect of stressors throughout the lifespan may play a significant role in shaping the mental health and well-being of young adults (Epel et al., 2018). In this model, lifetime stressor exposure refers to the combined and cumulative effect of stressors occurring over the entire lifespan, and includes acute life events (e.g., bereavement) and chronic difficulties (e.g., long-term financial issues problems; Mayer et al., 2019). This model is divided into three main components: (1) contextual factors (e.g., genetic influences), cumulative stressor exposure (e.g., distal and recent stressor exposure), and protective factors (e.g., family support); (2) psychophysiological stress responses (e.g., cardiovascular reactivity), and (3) biological ageing and disease (e.g., cardiovascular disease). The present study focuses specifically on the second component (i.e., psychophysiological responses) and the association between lifetime stressor exposure, mental health, and immunological responses. Contextual and protective factors, as well as long-term biological ageing and disease outcomes, are outside the scope of this study.

A central mechanism linking stress to health outcomes in this model is allostatic load, defined as the “wear and tear on the body and brain resulting from chronic overactivity or inactivity of physiological systems involved in adaptation to environmental challenge” (McEwen, 1998, p. 37). Repeated stressor exposure can dysregulate these systems, leading to maladaptive physiological states even when stressors remit, which over time contributes to disease onset and accelerated biological ageing (McEwen and Seeman, 1999; Ehlert et al., 2001). Accordingly, the integrative model suggests that cumulative stress exposure shapes psychophysiological responses, which in turn may lead to allostatic load and ultimately affect biological ageing and disease outcomes. Overall, this model suggests that exposure stressors can modify how individuals respond to future stressors (e.g., altered immune cell mobilisation), potentially leading to negative health outcomes (Epel et al., 2018).

To elaborate, exposure to stressors activates the hypothalamic-pituitary-adrenal (HPA) axis, formed of several structures, originating in the brain and ending in the adrenal glands. Acute stressors trigger a multisystem physiological response, comprising primarily the neuroendocrine and the immune system (Slavich et al., 2023). Neuroendocrine activation, specifically the release of adrenaline from the adrenal glands, mobilises immune cells into the bloodstream (Dhabhar et al., 2012). It is thought that the magnitude of this response represents immune system functioning, because these cells are subsequently redistributed from blood to peripheral tissues, searching for infected or damaged cells (Dhabhar et al., 2012). Indeed, this acute response to stress is thought to be part of a ‘fight or flight’ response, whereby the immune system is ‘primed’ to respond to threats. Importantly, chronic stress has been shown to significantly influence the mobilisation and function of immune cells. Prolonged exposure to stress hormones, such as glucocorticoids and catecholamines, can downregulate immune responses by altering the distribution, trafficking, and activity of various immune cells (Dhabhar, 2009). This dysregulation can contribute to immune suppression. Chronic stressor exposure can influence the functioning of immune cells (Cañas-González et al., 2020) and can impair aspects of immunity. Kiecolt-Glaser et al. (1987) found that caregivers had lower numbers of T cells, which are key immune cells in fighting infections, when compared to controls. It was also shown that caregivers exhibited a higher antibody response to viral exposure, possibly indicating poorer immune cell control of latent viruses. Similarly, Vedhara et al. (1999) reported that caregivers of relatives with dementia experienced greater emotional distress over a 28-day period compared with controls and this distress correlated with a weaker antibody response to the influenza vaccine.

However, the current knowledge of the association between lifetime stressor exposure, mental health and well-being (e.g., depression and anxiety), and immune cell mobilisation in response to acute stressors, is poorly understood (Epel et al., 2018). Most studies have examined the effect of acute stress on lymphocytes, which include T cells and Natural Killer cells that target infected or damaged cells, and B cells that produce antibodies. However, fewer studies have examined the effect of acute stress on other sub-types of immune cells, including monocytes and neutrophils, which act as a rapid first-line of defence, particularly against bacterial infections.

Despite evidence of the negative effects that stressor exposure can have on health, the multidimensional nature of lifetime stressor exposure is still poorly understood (Slavich and Shields, 2018). Research has focused on assessing the *frequency* of a limited number of stressors (e.g., serious accidents; Seery, 2013), as opposed to understanding the combined and cumulative effect of stressors over the lifespan (McLoughlin et al., 2021). Further research is needed that takes a more comprehensive, life-course approach, focusing on identifying and exploring the underlying dimensions of stressful exposures, such as their severity. Stressor severity represents a key dimension to measure given its contribution to the risk of physical ill-health and major illness (Boscarino, 2004; Vagg and Spielberger, 1999). Stressor severity is a marker of perceived impact, rather than the occurrence of the stressor (i.e., frequency; Arnold et al., 2024). This is important given that a measure of stressor occurrence would treat all stressors as equally impactful, whereas the perception of stressor impact can index important individual differences in stress responsivity and vulnerability (Shields et al., 2023).

To address these issues, the Stress and Adversity Inventory (STRAIN) has been developed to examine how lifetime stressor exposure is related to psychological, biological, and health outcomes (Slavich and Shields, 2018). Research using the STRAIN has found that greater lifetime stressor exposure is associated with more symptoms of depression and anxiety (e.g., Slavich et al., 2019), and more physical health complaints (e.g., Cazassa et al., 2020). Some of these chronic health conditions (e.g., metabolic syndrome and cardiovascular disease) have been shown to be related to systemic chronic inflammation, which is in-part induced by the activation of the immune system in the presence of certain social, psychological and environmental factors (e.g. stressors; Netea et al., 2017). Furthermore, lifetime stressor exposure may degrade health, when demands are chronic (i.e., stressors that are demanding, distressing, and on-going for six months or more), have occurred more recently (i.e., in adulthood, encompassing all stressors experienced after the age of 18; McLoughlin et al., 2021), or are perceived as more severe (i.e., how “stressful” it is deemed for an individual; McLoughlin et al., 2022a). However, researchers have yet to examine how lifetime stressor exposure relates to the mobilisation of the main immune cell sub-types (e.g., lymphocytes, monocytes and neutrophils) in response to an acute stressor (e.g., speech task), and to consider its relationship with mental health and well-being. This is important given that aspects of immunity are objective markers of health and therefore this work will provide insight to relationships between lifetime stressors and these immune markers of health. To date, no study has simultaneously examined lifetime stressor exposure, mental health, well-being, and immune mobilisation within a single integrative model, nor used network-based approaches to characterise the multivariate relationships among these domains.

To address these issues, we examined how lifetime stressor exposure (i.e., frequency and severity) relates to mental health, well-being, and immune cell mobilisation in response to an acute social stressor. Based on the integrative model of lifespan stress and health, we formulated three hypotheses: (1) Lifetime stressor exposure and mental health: Higher frequency and severity of lifetime stressor exposure will be positively associated with symptoms of depression and anxiety. (2) Mental health and immune mobilisation: General lower well-being will be associated with smaller immune cell mobilisation (lymphocytes and monocytes) following the acute social stressor. (3) Network structure: Variables reflecting stressor severity and psychological symptoms will emerge as central nodes within the network, given their established role in stress-related psychophysiological dysregulation and their expected associations with well-being and immune mobilisation.

To test these novel research questions and hypotheses, a subset of data were re-analysed from a previously published study (i.e., McLoughlin et al., 2022b). The original study focused on lifetime stressor exposure, cardiovascular responses, and cortisol reactivity to acute social stress. In contrast, the present study addresses a distinct research question, examining the association between lifetime stressor exposure, mental health, well-being, and immunological responses. While all participants, procedures, and baseline measures were collected as described in McLoughlin et al. (2022b), the current analyses include novel variables, specifically immune cell mobilisation and mental health and well-being outcomes, which were not previously analysed or reported in the original publication. This distinction ensures that the current manuscript provides new insights beyond the scope of the original study. Any analyses reported here were not included in the original study.

## 2 Method

### 2.1 Participants

Participants were recruited as reported in McLoughlin et al. (2022b). The original sample included 86 participants (45 female, 41 male; *M*_*age*_ = 23.31 years, *SD* = 4.94). Exclusion criteria were identical to the original study and included: known respiratory or cardiovascular conditions, a history of diabetes, were currently ill, injured, or pregnant, were at high risk for infection, or were taking medications that could increase infection risk (e.g., steroids). Additional exclusion criteria included smoking (defined as consuming at least one cigarette per day) and obesity (defined as a body mass index >30 kg/m^2^). An a priori power analysis conducted using G∗Power software for the original study indicated that a minimum sample size of 77 participants was required to perform multiple regression analyses. The calculation was based on a medium effect size (β = 0.30) found between lifetime stressor exposure and cortisol reactivity in Lam et al. (2019), with an alpha level of 0.05 and a desired power of 0.80. This power estimate is consistent with previous published research employing similar analytical approaches. Moreover, drawing on Lakens’ (2022) discussion of heuristics and rules of thumb for sample size justification, it is worth noting that comparable studies using a network analysis have typically adopted similar sample sizes (e.g., Varol et al., 2023), further supporting the adequacy of the sample size used in the present study.

### 2.2 Procedure

The study received institutional ethical approval from the University of Bath (EP 19/20 027). Participants were recruited through convenience and volunteer sampling, primarily via the researchers’ existing networks. The sample was predominantly composed of university students, with recruitment conducted across various campus settings, including lecture halls, student common areas, and online student mailing lists, allowing us to reach a convenience sample of volunteers. Prior to participation, individuals received an information sheet outlining their ethical rights and provided written informed consent. Participants then completed a 20-min questionnaire, which assessed lifetime stressor exposure, symptoms of depression and anxiety, and well-being. This questionnaire was completed at least 48 h before the laboratory session to prevent any potential influence on acute stress responses. Laboratory sessions were scheduled between 13:00 and 18:00 and lasted approximately 3 h. To minimize the impact of external factors on immune cell data, participants were instructed to abstain from caffeine, alcohol, and moderate-to-vigorous exercise for 24 h prior to their laboratory session. Participants were asked to refrain from eating or drinking anything other than water for 4 h before their visit. Upon arrival, participants rested for 20 min before providing a finger-tip blood sample. They were then briefed on the upcoming laboratory-based social stress test (Labuschagne et al., 2019). After a 5-min preparation period, participants completed a modified version of the Trier Social Stress Test (TSST). This was immediately followed by another finger-tip blood sample (see Supplementary Materials for an illustration of the protocol).

The modified version of the TSST has been extensively validated (Labuschagne et al., 2019) and includes a 5-min speech task, followed by a 5-min mental arithmetic task. For the speech task, participants had 5 min to prepare and then 5 min to deliver a speech to “senior management” describing why they would be “the perfect applicant for the vacant position”. They were told their speech would be videotaped “so that a video analysis of behaviours, voice frequency, and performance may be conducted.” Following this, participants were asked to complete a 5-min mental arithmetic task in which they started at 1022 and “serially subtracted 13 as fast and as accurately as possible.” Errors, slow counting, or hesitation required them to restart from the beginning. While the procedures were conducted as described in the original study (i.e., McLoughlin et al., 2022b), the analyses presented here represent a novel examination of the associations between lifetime stressor exposure, mental health, well-being, and immune cell mobilisation in response to an acute social stressor.

### 2.3 Measures

#### 2.3.1 Lifetime stressor exposure

Lifetime stressor exposure was assessed using the Adult STRAIN (Slavich and Shields, 2018). This is a comprehensive tool that measures 55 significant life stressors including 26 acute life events (e.g., bereavement) and 29 chronic difficulties (e.g., ongoing financial difficulties). When a participant reports experiencing a stressor, they respond to follow-up questions assessing: perceived severity (*“At its worst, how stressful/threatening was this demand for you?“*, rated from 1 = not at all to 5 = extremely), frequency (*“How many times have you experienced this stressor?“*, ranging from once to five or more times), timing of exposure (*“When did this happen?“*, ranging from 1 = ongoing to 7 = more than five years ago), and duration (*“How long did you experience this for?“*, recorded in months and/or years). For analysis, the following variables were used: (a) total count of early life stressors, (b) total severity of early life stressors, (c) total count of adulthood stressors, and (d) total severity of adulthood stressors. Higher scores indicate greater exposure to lifetime stress. The Adult STRAIN has demonstrated excellent test-retest reliability (rs = 0.90–0.95) and strong concurrent (rs = 0.15–0.62) and predictive validity across various mental, physical, and biological health outcomes (Cazassa et al., 2020).

#### 2.3.2 Depression

Depressive symptoms over the previous two weeks were assessed using the Patient Health Questionnaire (PHQ-9; Kroenke et al., 2001). This measure consists of nine items (e.g., “little interest or pleasure in doing things”), each rated on a 4-point Likert scale from 0 (not at all) to 3 (nearly every day). Scores range from 0 to 27, with higher totals indicating more severe depressive symptomatology. Cut-off scores of 5, 10, 15, and 20 denote mild, moderate, moderately severe, and severe levels of depression, respectively (Kroenke et al., 2001). The PHQ-9 has demonstrated strong internal consistency (α = .86–0.89), high test-retest reliability (r = 0.84), and solid construct and criterion validity. In the current study, internal consistency was acceptable (α = .82).

#### 2.3.3 Anxiety

Anxiety symptoms experienced over the past two weeks were measured using the Generalised Anxiety Disorder scale (GAD-7; Spitzer et al., 2006). This instrument comprises seven items (e.g., feeling nervous, anxious, or on edge), each rated on a 4-point Likert scale from 0 (not at all) to 3 (nearly every day). Total scores range from 0 to 21, with higher scores reflecting more severe anxiety symptoms. Thresholds of 5, 10, and 15 correspond to mild, moderate, and severe anxiety, respectively (Spitzer et al., 2006). The GAD-7 demonstrates excellent internal consistency (α = .89–0.92), strong test-retest reliability (rs = 0.83), and robust evidence of convergent, construct, criterion, and factorial validity. In the present study, internal consistency was high (α = .88).

#### 2.3.4 Psychological well-being

Well-being over the past two weeks was measured using the WHO Well-Being Index (WHO-5; WHO, 1998). This scale includes five items (e.g., “I have felt cheerful and in good spirits”), each rated on a 6-point Likert scale from 0 (at no time) to 5 (all the time). The total raw score (range = 0–25) was multiplied by 4 to yield a final score ranging from 0 to 100, with higher scores indicating greater well-being. Scores below 50 are indicative of poor well-being. The WHO-5 has demonstrated excellent internal consistency (α = .90) as well as strong construct and convergent validity (Topp et al., 2015). In the present study, the WHO-5 showed good internal consistency (α = .80).

#### 2.3.5 Capillary blood sampling

Capillary blood sampling was performed by skin puncture at the fingertip. Throughout data collection, different fingers were used for each time point (Pre- and Post-TSST) within the same participant to avoid confounding by an acute inflammatory response to previous skin punctures. However, for consistency, the same fingers (e.g., index finger, middle finger) were used in the same order for both time points across participants. Approximately 200 μL of blood was collected into tubes (MiniCollect 0.25/0.5 mL K3EDTA; Greiner BioOne, Kremsmunster, Austria), and a three-part leukocyte differential was assessed immediately using an automated haematology analyser (Sysmex KX-21N, Norderstedt, Germany). The following variables were used in analyses: absolute counts of cells expressed × 10^9^/L of blood: lymphocytes, monocytes and neutrophils for descriptive and comparative purposes. Values for monocytes were derived from the “mixed cells” fraction from the automated haematology analyser (<10 % are basophils and eosinophils). For the network analysis, immune responses were represented as difference (Δ) scores from Pre-to Post-TSST (Δ-TSST), as these provide a direct index of stress-induced immune mobilisation. Because change scores already incorporate baseline values, baseline counts were not included separately.

### 2.4 Data analysis

After calculating descriptive statistics, a series of paired samples *t*-tests were conducted to assess the changes in lymphocyte, monocyte, and neutrophil count over time. These analyses confirmed that the TSST caused an acute immune response to stress. Prior to formal analysis, absolute change in immune cell (lymphocytes, monocytes and neutrophils) count in response to stress was calculated by subtracting baseline values (i.e., first finger-tip blood sample) from stressor exposure values (i.e., second finger-tip blood sample). To control for multiple testing and manage the rate of type I errors, the False Discovery Rate (FDR) correction was performed using the Benjamini-Hochberg procedure, with statistical significance defined as an FDR-corrected P-value (q-value) ≤ 0.05 (Benjamini and Hochberg, 2000). Only the variables that were statistically significant (FDR-adjusted p-value ≤0.05) were used for the subsequent analysis.

Associations between the variables were assessed via network analysis. In a network analysis, variables are considered as nodes, connected by edges that represent the relationships between nodes. In this study, a Gaussian Graphical Model (GGM) and a Bayesian Network (BN) were constructed to provide complementary information on the relationships between variables. GGMs are undirected networks where edges represent pairwise conditional dependencies (Lauritzen, 1996), and they have been used to analyse complex relationships among psychological (Pino et al., 2020) and biological (Shutta et al., 2022) data. In GGMs, edges can be weighted to represent partial correlations between variables. BNs provide complementary information by indicating the direction of the edges, allowing for the interpretation of relationships within a causal cascade (Briganti et al., 2022). In this study, psychological and immunological variables were considered as nodes of a GGM and BN. The GGM was estimated by searching for the optimal model, refitting models constructed through graphical Least Absolute Shrinkage and Selection Operator (LASSO; Friedman et al., 2008) and minimising the Extended Bayesian Information Criterion (EBIC), as it has been shown to converge to a true model (Foygel and Drton, 2010). This involved selecting the best model and modifying edges until the EBIC could no longer be improved (Foygel and Drton, 2010).

Network centrality statistics were calculated to assess node relationships, specifically: (1) strength (i.e., sum of weights between a node and other connected nodes); (2) closeness (i.e., average length of the shortest path between a node and any other node); and (3) betweenness (i.e., how many times a node serves as a “bridge” on the shortest path between two other nodes; Opsahl et al., 2010). Then, 95 % Confidence Intervals (95 % CIs) were estimated for each statistic by conducting 1000 non-parametric bootstraps (Epskamp et al., 2018). Given this theoretical focus, strength centrality is the most appropriate measure for addressing our research aims, whereas Expected Influence would reflect a different conceptual question centred on net directional influence. Moreover, as our variables were conceptualised as components of a single integrated system rather than opposing functional processes or distinct symptom communities, measures such as Expected Influence or bridge centrality were not theoretically justified or necessary for the interpretation of the present network. In this study, sample centrality values were therefore used for interpretation, while bootstrapped values were used solely to assess stability. The Bayesian network estimated was a Directed Acyclic Graph (DAG). To estimate the DAG, a score-based structure learning algorithm was used, specifically, the Hill-Climbing greedy search algorithm (Russell and Norvig, 2010). This process involved 50 random restarts, with each restart undergoing 100 perturbations, and the Bayesian Information Criterion (BIC) was used to evaluate model fit. Further, a bootstrapping approach was performed, generating a set of 1000 networks. Statistically significant edges were identified based on an empirical threshold derived from the average of the bootstrapped networks and the directionality of the significant edges was determined by the most frequent orientations observed in the bootstrapped networks (Scutari & Nagarajan, 2013). The empirical threshold refers to a data-driven cutoff estimated from the distribution of bootstrap edge confidences, following Scutari and Nagarajan (2013). Instead of selecting an arbitrary value (e.g., 0.8), this method derives the cutoff that best separates low-confidence from high-confidence edges. Strengths of our approach include it being data-driven, and the two approaches offering complementary information. Indeed, the GGM highlights the positive and negative relationships between variables and provides centrality statistics, while the DAG illustrates cascade effects between variables and the likely direction of these relationships. The GGM and DAG were constructed and visualised using the qgraph (Epskamp et al., 2017), bootnet (Epskamp and Fried, 2015), Rgraphviz (Hansen et al., 2015), and bnlearn (Scutari et al., 2019) packages in R version 4.3.3.

## 3 Results

### 3.1 Descriptive statistics

Participants experienced an average of 17 stressors over their lifetime (range = 0–54), and the mean cumulative lifetime stressor severity was 27.07 (*SD* = 17.70; range = 0–265). On average, participants reported mild symptoms of depression (*M* = 5.08, *SD* = 4.34), minimal symptoms of anxiety (*M* = 4.88, *SD* = 4.24), and relatively high well-being (*M* = 60.79, *SD* = 15.54). Overall, 1.2 %, 2.3 %, 12.8 %, and 29 % of participants met the criteria for severe, moderately severe, moderate, and mild depression, respectively. In addition, 2.3 %, 10.5 %, and 27.9 % of participants met the criteria for severe, moderate, and mild anxiety, respectively. Finally, 24.4 % of participants scored <50 on the WHO-5, indicating poor well-being.

### 3.2 Immune cell mobilisation in response to the TSST

A paired samples *t*-test showed a statistically significant difference in lymphocyte cell count (*t*(85) = −7.041, *p* < .001; FDR-adjusted *p* < .001), with levels significantly higher immediately after the TSST (*M =* 2.32, *SD* = 0.60) compared to baseline (*M* = 2.07 × 10^9^/L blood, *SD* = 0.50). Additionally, a paired samples *t*-test showed a statistically significant difference in monocyte cell count (*t*(85) = −6.175, *p* < .001; FDR-adjusted *p* < .001), with levels significantly higher immediately after the TSST (*M =* 0.66 × 10^9^/L blood, *SD* = 0.22) compared to baseline (*M* = 0.55 × 10^9^/L blood, *SD* = 0.16). A paired samples *t*-test initially showed a statistically significant difference in neutrophil cell count, with levels slightly higher after the TSST (M = 4.37 × 10^9^/L blood, SD = 1.39) compared to baseline (M = 4.25 × 10^9^/L blood, SD = 1.37; t(85) = −2.600, *p* = .011); however, this difference was no longer significant after FDR correction (FDR-adjusted *p* = .056). As a result, neutrophil data were excluded from the subsequent analysis.

### 3.3 Primary analyses

**Fig. 1:**
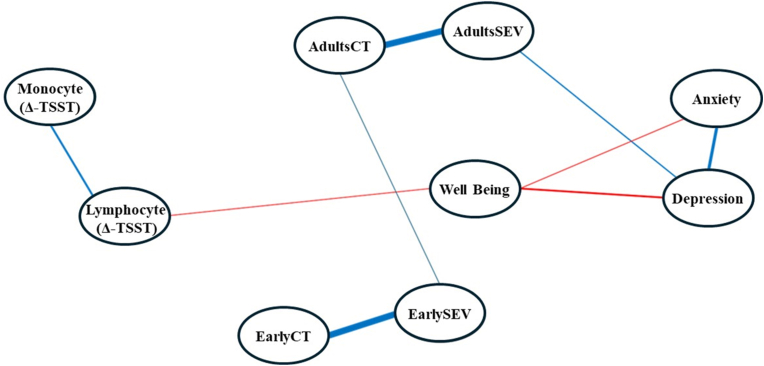
The GGM resulted from the analysis. Blue lines represent positive partial correlations, while red lines represent negative partial correlations Please Note: AdultCT = Total count of adulthood stressors; AdultSEV = Total severity of adulthood stressors; EarlyCT = Total count of early life stressors; and EarlySEV = Total severity of early life stressors. Node positions are determined by the Fruchterman–Reingold layout for visual clarity and do not represent statistical or conceptual distances. (For interpretation of the references to colour in this figure legend, the reader is referred to the Web version of this article.)

**Fig. 2:**
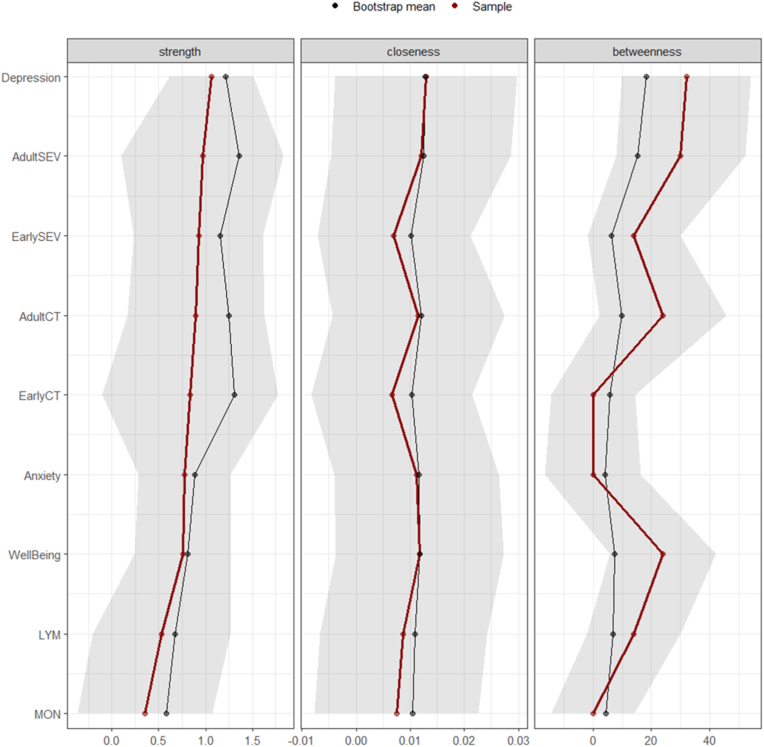
GGM centrality measures from the original sample and bootstrap results. The interpretation of central nodes is based on the sample centrality values. **Please Note:** AdultCT = Total count of adulthood stressors; AdultSEV = Total severity of adulthood stressors; EarlyCT = Total count of early life stressors; and EarlySEV = Total severity of early life stressors; LYM = lymphocyte mobilisation; and MON = monocyte mobilisation.

The GGM from the analysis is depicted in Fig. 1 and the adjacency matrix is reported in Table 1. Centrality statistics and their 95 % CIs are reported in Fig. 2. The interpretation of central nodes is based on the sample centrality values. The GGM analysis indicated that symptoms of depression, total count and severity of adulthood stressors, and well-being were among the most strongly connected variables in the network. Symptoms of depression, severity of adulthood stressors, and severity of childhood stressors exhibited the highest strength values (Fig. 2), indicating their significant direct relationship with other variables (e.g., the depression node has the highest strength value compared to other nodes in the network constructed from the sample), and a central role in the network relationships. When considering symptoms of depression, its highest strength value is related to connections with total severity of adulthood stressors, symptoms of anxiety, and well-being. This suggests that depression is not only a central node but also occupies an intermediary position among these key variables within the network (e.g., well-being and the severity of adult stressor, respectively). Moreover, symptoms of depression, the severity of adulthood stressor nodes along with the total count of adulthood stressors, and well-being demonstrated high betweenness values (Fig. 2), suggesting their importance as bridges connecting various nodes. Indeed, symptoms of depression, total count and severity of adulthood stressors, and well-being serve as essential bridges, connecting different nodes within the network. In light of these results, changes in symptoms of depression, the total count or severity of adulthood stressors, the severity of childhood stressors, or well-being were likely to exhibit notable centrality within the network. The centrality statistics and network representation in Fig. 1 suggests that more frequent and severe stressors experienced during early life are linked to more frequent and severe stressors experienced in adulthood, which are associated with mental ill-health (e.g., depressive symptoms), and ultimately smaller immune responses (e.g., immune cell mobilisation) to acute stress. This relationship explains the higher statistics observed for strength and betweenness.

**Table 1:** Adjacency matrix of the GGM with the weight between pairs of nodes. Partial correlations are regularised estimates from the graphical LASSO procedure represent non-zero partial correlations that remain after regularisation.

	Well-Being	Depression	Anxiety	Lymphocyte	Monocyte	Total Count of Early Stressors	Total Severity of Early Stressors	Total Count of Adult Stressors	Total Severity of Adult Stressors
Well-Being	1								
Depression	−0.36	1							
Anxiety	−0.23	0.56	1						
Lymphocyte	−0.17	0	0	1					
Monocyte	0	0	0	0.36	1				
Total Count of Early Stressors	0	0	0	0	0	1			
Total Severity of Early Stressors	0	0	0	0	0	0.84	1		
Total Count of Adult Stressors	0	0	0	0	0	0	0.09	1	
Total Severity of Adult Stressors	0	0.16	0	0	0	0	0	0.81	1

### 3.4 Results obtained from Directed Acyclic Graph (DAG)

**Fig. 3:**
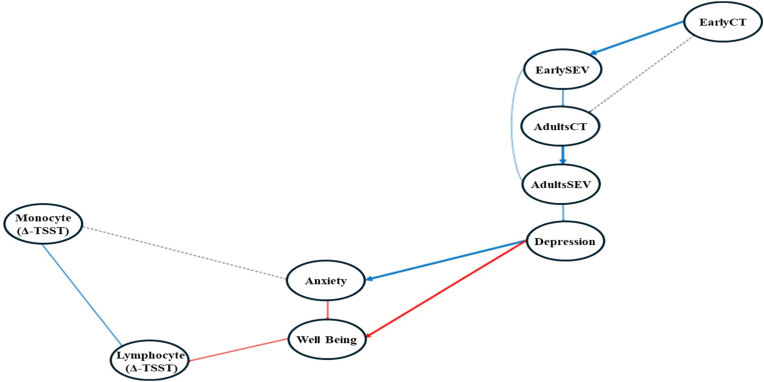
DAG results: The thickness of the lines depicts the strength (an edge probability for inclusion) of the significant relationships, while dashed lines indicate non-significant relationships. Please Note: AdultCT = Total count of adulthood stressors; AdultSEV = Total severity of adulthood stressors; EarlyCT = Total count of early life stressors; and EarlySEV = Total severity of early life stressors. The layered layout is used for visual clarity only; node positions do not carry statistical meaning. Only the arrows and their direction represent conditional associations.

Additional and complementary information is provided by the DAG model. Indeed, the network structure obtained from the DAG confirmed the model derived from the GGM, as this distinct data-driven approach revealed similar connections between the nodes (Fig. 3). This indicates a convergence of the results. Additionally, the cascade visualisation provides further insights into the model. Specifically, exposure to more frequent and severe stressors during early life may lead to more frequent and severe stressors during adulthood, which increased symptoms of depression that could, in turn, affect symptoms of anxiety. Overall, the DAG illustrates how exposure to more frequent stressors in early life may lead to individuals experiencing more frequent and severe stressors in adulthood, subsequently increasing symptoms of depression and anxiety, which is then associated with higher symptoms of depression and anxiety. These symptoms are in turn linked to lower well-being, which is associated with smaller immune cell mobilisation in response to the acute stressor.

## 4 Discussion

Recently, there has been an increase in research examining factors that influence the mental health and well-being of young adults (Wyn, 2022). Although exposure to stressors may be an important factor in the mental health and well-being of young adults (Slavich et al., 2019), researchers have yet to examine the impact of lifetime stressor exposure on immune cell mobilisation to acute stressors. Understanding this relationship is important, as frequent or severe lifetime stressors may contribute to maladaptive physiological responses, potentially increasing susceptibility to negative health outcomes (McLoughlin et al., 2022b). Stress is a ubiquitous, evolutionarily conserved process that activates multiple physiological systems to enhance performance, promote adaptation, and protect the body from harm (Dhabhar, 2014). Under optimal conditions, these responses are short-lived, with physiological systems rapidly activating and deactivating to restore the body to a steady state (Turner et al., 2020). However, when stress becomes chronic or prolonged, these same systems can become dysregulated (McEwen, 1998). Short-term stress is generally immuno-enhancing, involving changes in dendritic cell, neutrophil, macrophage, and lymphocyte trafficking, maturation, and cytokine production (Dhabhar, 2014). In contrast, chronic stress is immunosuppressive, disrupting both innate and adaptive immune responses (Dhabhar, 2019).

Our findings suggest that lifetime stressor exposure may contribute to this physiological dysregulation. Specifically, results revealed that more frequent and severe stressors experienced during early life rendered individuals more susceptible to stressors during adulthood, which were positively associated with symptoms of depression and subsequent anxiety. This mental ill-health symptomology then deterred well-being, which was associated with a small magnitude of immune cell mobilisation to the acute stressor. These findings support aspects of the integrative model of lifespan stress and health (Epel et al., 2018), highlighting how greater exposure to lifetime stressors may influence mental health and well-being, as well as immunological responses to acute stress. This suggests that lifetime stressor exposure may contribute to allostatic load, reflecting the wear-and-tear on the body and immune system that can arise from repeated or severe stress, which over time may increase vulnerability to mental and physical health problems (McEwen, 1998; McEwen and Seeman, 1999). However, contextual factors, protective factors, and long-term biological ageing or disease outcomes were not assessed in this study but should be the subject of future research.

The results indicate that exposure to more frequent and severe stressors over the lifespan operates through mental ill-health and well-being, which in turn, affects the magnitude of immune cell mobilisation in response to an acute psychological stressor. This is consistent with prior research which highlights that greater stressor exposure increases the incidence of depressive symptoms (Fried et al., 2015). In fact, stressors are associated with neurodevelopmental changes (e.g., cognitive control) that relate to mood and anxiety disorders (Syed and Nemeroff, 2017) and represent a risk factor for depression and anxiety (Dyson and Renk, 2006). These two symptoms are closely connected as they are both internalising disorders and are highly comorbid (Kalin, 2020). Within this study, symptoms of anxiety and depression were related to well-being, which have been linked negatively with aspects of immune function (i.e., lymphocyte mobilisation in response to acute stress (i.e., TSST). Although there is extensive recognition of the impact of lifetime stressors on symptoms of depression and anxiety (Lähdepuro et al., 2019), our findings highlight the relationship between lifetime stressor exposure and immune cell mobilisation in response to acute stress. While it is known that stressful situations can negatively impact well-being (Savage et al., 2024), the two networks also provide further insight into how exposure to early life stressors (e.g., abuse, neglect) are associated with poorer well-being (McElroy and Hevey, 2014). This association is based on a cascade effect, explaining the association between early life stressors and mental ill-health and poorer well-being. In the models, both depressive and anxiety symptoms had a direct negative impact on well-being (Stein and Heimberg, 2004), which negatively affected lymphocyte mobilisation to the acute stressor. Thus, the models suggest that the response of lymphocytes to an acute psychological stressor is not directly associated with depression and anxiety symptoms, but with the broader dimension of well-being passing through symptoms. Supported by previous literature, our results provide further evidence linking well-being with immune system functioning (Lasselin et al., 2016).

Results highlight the relationship between early life stressor exposure, well-being, and lymphocyte mobilisation in response to an acutely stressful situation. This finding is important as it suggests that the well-being of young adults may have an impact on aspects of immune function in response to socially stressful situations, an event many young adults may encounter (Koudela-Hamila et al., 2022). A lowered lymphocyte and monocyte concentration in those experiencing early life stressor as suggested in this study, could mean these individuals have a lowered ability to fight infections. Both these cell types are important in our immune responses to infection risk as the monocyte population form part of our innate immune system and the lymphocytes form part of the adaptive immune system. Both are important elements of a response to infection. It is well-documented that exposure to psychological stress during early years can have a negative impact on the immune system extending into adulthood (Fagundes and Way, 2014). Previous research has highlighted that psychological stress in children may alter aspects of the immune response (Carlsson et al., 2014). To elaborate, exposure to significant stressors during childhood can lead to impaired lymphocyte responses to pathogenic stimuli (Carlsson et al., 2014). Furthermore, exposure to frequent and severe stressors can increase pro-inflammatory responses to immune challenges reported in this population which might drive auto-immunity or chronic disease linked to inflammation (Copeland et al., 2014) such as cardiovascular disease and type 2 diabetes (Berbudi et al., 2020). Taken together, these findings suggest that repeated exposure to significant stressors during childhood may contribute to allostatic load, reflecting the cumulative wear-and-tear on the body and immune system. This aligns with the broader conceptualization that chronic or repeated stress can dysregulate physiological systems over time, potentially increasing vulnerability to mental and physical health problems later in life (McEwen, 1998; McEwen and Seeman, 1999).

The results of the present study indicated a strong, positive relationship between lifetime stressor frequency (i.e., total count of adulthood stressors and total count of early life stressors) and severity (i.e., total severity of early life stressors and total severity of adulthood stressors). Indeed, the results suggest that prior exposure to stressors is associated with the perceived impact of subsequent stressors (i.e., how ‘stressful’ a new demand is judged to be for an individual). This is particularly important given the deleterious health consequences associated with experiencing severe stressors (e.g., Cazassa et al., 2020; Slavich et al., 2019). As an example, McLoughlin and colleagues' (2022a) found that the total severity of lifetime stressors experienced by sport performers predicted symptoms of depression and anxiety, physical health complaints, and well-being. One theoretical model that may help explain this finding is the biopsychosocial model (BPSM) of challenge and threat (Blascovich, 2008). According to this model, individuals are more likely to perceive an acutely stressful situation as threatening when their available coping resources are insufficient. This study extends existing work by highlighting the strength and direction of the relationship between lifetime stressor frequency and severity.

Building on this viewpoint, the biopsychosocial model (BPSM) of challenge and threat (Blascovich and Tomaka, 1996; Mendes and Park, 2014) offers a helpful lens for explaining why people vary, both between and within individuals, in their reactions to stress. The model proposes that people differ in how they interpret stressful situations and in the physiological patterns that accompany those interpretations. When someone believes they have the skills and resources to handle a demanding situation, they experience it as a challenge. In contrast, when they judge that the situation's demands outweigh their coping capacity, they experience it as a threat (Blascovich, 2008). According to the BPSM, a challenge state produces a cardiovascular profile associated with “physiological toughness,” marked by increases in heart rate and cardiac output alongside reduced total peripheral resistance. A threat state, however, is linked to a “physiological weakness” pattern in which heart rate rises, cardiac output shows minimal change or decreases, and total peripheral resistance stays the same or increases (Seery, 2011). Threat appraisals have been linked to less adaptive emotional, motivational, neuroendocrine, autonomic, and behavioural outcomes (Blascovich and Mendes, 2000), and have even been related to biological indicators of accelerated ageing (e.g., shorter telomere length; O'Donovan et al., 2012). Distinguishing between challenge and threat offers researchers a way to understand why identical stressors can be harmful for some individuals yet not for others, and how certain stressors may even yield positive effects (Seery et al., 2010). It is possible that lifetime stressor exposure shapes how individuals appraise subsequent stressors over time. Specifically, greater exposure to stressors may lead individuals to interpret stressors as a threat, which have in turn been linked to poorer mental health and well-being, as well as markers of cellular ageing (Mendes and Park, 2014; O'Donovan et al., 2012). Heightened threat appraisals could represent one mechanistic pathway linking stressor exposure to both poorer psychological outcomes and lower lymphocyte count. This highlights the importance of resilience and coping processes as potential protective factors that may buffer the impact of cumulative stress on both psychological and immunological health.

Our results suggest that exposure to a high number of stressors during early life might lead to more frequent and severe stressors being reported in adulthood, which may have deleterious consequences for health and well-being and aspects of immune system functioning. Thus, practitioners working with children could use these findings to identify, and provide tailored support to, children who are at-risk of developing stress-related health problems (e.g., those currently experiencing a high number of or severe stressors in their early life). This is important given that early intervention can prevent and reduce the progression of mental illness, alleviate suffering, and improve long-term health (e.g., Schinke et al., 2018). Therefore, practitioners could reduce the stressors children experience by modifying the environment in which they reside. Given that it is not always possible nor desirable to reduce or eliminate stressors, however, practitioners should also work with young adults to ensure that they better respond to and manage stressors (Fletcher and Arnold, 2021). One intervention that might be useful is the stress optimisation approach (Crum et al., 2020), which encourages individuals to view stress responses as beneficial (e.g., increased heart rate). Indeed, research has shown that fostering a stress-is-enhancing mindset and prompting reappraisal of stress-induced physiological arousal can improve health and well-being (Jamieson et al., 2018).

Several strengths of this study should be noted. This study provides a comprehensive examination of the relationships between lifetime stressor exposure (i.e., frequency and severity), mental ill-health and well-being related variables (e.g., symptoms of depression), and immune system functioning (e.g., lymphocyte mobilisation) to a laboratory-based social stressor. Next, analysing the data using a network analysis provides a powerful tool for understanding and leveraging the intricate web of associations and interactions within the variables of interest (Shirinivas et al., 2010). Indeed, our analytical approach enabled us to identify influential nodes in the network, such as total count of early life stressors, which can be important for understanding the interplay between variables (Borsboom and Cramer, 2013). This will lead to more informed decision-making and innovative solutions (e.g., development of effective interventions; Vagnetti et al., 2020). Additionally, the strength of the network analysis approach lies in its data-driven nature, ensuring that the results were not restricted by assumptions. Finally, this study has been conducted with the Integrative Model of Lifespan Stress and Health considered in its formation, implementation, and interpretation (Epel et al., 2018). This is particularly beneficial given that the integrative model captures the overall stress process, utilising a multidimensional and interdisciplinary perspective to better understand the relationships between lifetime stressor exposure and health.

Notwithstanding these strengths, several limitations should be noted. First, only a relatively limited number of objective markers were assessed. The measurement of immune cells in the current study represents a single snapshot of the response to an acute stressor, which may not fully capture the dynamics of immune mobilisation. This is important as assessing additional objective markers can provide a more comprehensive understanding of the relationships between lifetime stressor exposure, mental ill-health and well-being, and immune cell mobilisation. Second, our assessment of lifetime stressor exposure relied on retrospective self-report, which can be susceptible to recall bias. Current emotional states, such as depression or anxiety, may influence how participants remember or evaluate past events, potentially inflating associations between stress exposure and mental health outcomes. Although self-report checklist measures are relatively inexpensive and easy to administer, concerns have been raised regarding their reliability and validity (Dohrenwend, 2006). Nevertheless, the STRAIN has demonstrated excellent test-retest reliability (Cazassa et al., 2020) and individuals can reliably recall major life stressors over long periods (Slavich and Shields, 2018). Next, participants in this study were predominantly young, with an average age of 23, which may have constrained the diversity in both the frequency and intensity of stressors encountered. Additionally, our sample was recruited using convenience and volunteer sampling from university settings, which may introduce selection bias and limit the generalizability of the findings to broader populations. Another potential limitation of the current study is that individuals with known illnesses, smokers, and those with obesity were excluded. While this approach was necessary to minimize confounding effects on immune measures, it may limit the generalizability of our findings to the broader population, particularly to individuals with these characteristics. Therefore, future research should explore the applicability of these findings across a broader and more varied population (James et al., 2019). The study did not control for additional factors that may influence psychological or immune responses, such as alcohol consumption, sleep quality, age-related differences, or gender-related variation. Future research would benefit from examining these variables more comprehensively. Finally, researchers should consider collecting data over time to better capture the on-going nature of lifetime stressors and their causal associations with key outcomes (Charles et al., 2013). Relationships in this study should be interpreted with caution, as while the DAG indicates the direction of the relationships, the cross-sectional design of the study limits the ability to infer causality. As a result, future research should adopt a longitudinal design to allow for a more nuanced and comprehensive understanding of how young adults respond to the stressors encountered at different time points, and how this subsequently impacts health.

In conclusion, this study examined the relationships between lifetime stressor exposure (i.e., frequency and severity), mental ill-health and well-being (e.g., symptoms of depression), and immune cell mobilisation (e.g., lymphocytes) to a laboratory-based social stressor in young adults. Using a network analysis approach, the results suggested that experiencing more frequent and severe stressors during early life may have increased susceptibility to experiencing more frequent and severe stressors during adulthood, which, in turn, might have increased vulnerability to mental ill-health (i.e., more symptoms of depression and subsequent anxiety). Additionally, poorer mental health was linked to lower well-being, which reduced lymphocyte mobilisation to the acute stressor. These findings suggest that repeated exposure to stressors across the lifespan may contribute to allostatic load, reflecting physiological wear-and-tear that can influence long-term health outcomes. As a result, this study advances prior research by incorporating a multidimensional and interdisciplinary perspective to better understand the relationships between lifetime stressor exposure and mental ill-health. The results highlight the importance of assessing lifetime stressor exposure for researchers and clinicians aiming to study the social-environmental drivers of poor immune and clinical health, as well as for identifying individuals at risk of accumulating allostatic load and subsequent stress-related disease.

## CRediT authorship contribution statement

**Ella McLoughlin:** Writing – original draft, Visualization, Project administration, Methodology, Investigation, Formal analysis, Data curation, Conceptualization. **Daniele Magistro:** Writing – review & editing, Visualization, Supervision, Formal analysis, Data curation, Conceptualization. **Roberto Vagnetti:** Writing – original draft, Visualization, Formal analysis, Data curation, Conceptualization. **George M. Slavich:** Writing – review & editing, Visualization, Supervision, Resources. **James E. Turner:** Writing – review & editing, Visualization, Supervision, Methodology, Investigation, Data curation, Conceptualization. **Rachel Arnold:** Writing – review & editing, Visualization, Supervision, Methodology, Investigation, Conceptualization. **Lee J. Moore:** Writing – review & editing, Visualization, Supervision, Methodology, Investigation, Conceptualization. **John Hough:** Writing – original draft, Visualization, Supervision, Formal analysis, Data curation.

## Funding

G.M.S. was supported by grant #OPR21101 from the California Governor’s Office of Planning and Research/California Initiative to Advance Precision Medicine. This work was supported by the EPSRC -NIHR funds (grant number EP/W031809/1, IMACTIVE). The findings and conclusions in this article are those of the authors and do not necessarily represent the views or opinions of these organisations, which had no role in designing or planning this study; in collecting, analysing, or interpreting the data; in writing the article; or in deciding to submit this article for publication.

## Declaration of competing interest

The authors declare that they have no known competing financial interests or personal relationships that could have appeared to influence the work reported in this paper.

## Data Availability

Data will be made available on request.

## References

[bib1] Arnold R., Brown D.J., McLoughlin E. (2024). An examination of the relationship between sport performers' organizational stressor dimensions, physical health, and well-being. J. Sports Sci..

[bib2] Benjamini Y., Hochberg Y. (2000). On the adaptive control of the false discovery rate in multiple testing with independent statistics. J. Educ. Behav. Stat..

[bib3] Berbudi A., Rahmadika N., Tjahjadi A.I., Ruslami R. (2020). Type 2 diabetes and its impact on the immune system. Curr. Diabetes Rev..

[bib4] Blascovich J., Elliot A.J. (2008). Handbook of Approach and Avoidance Motivation.

[bib5] Blascovich J., Mendes W.B., Forgas J.P. (2000). Feeling and Thinking: the Role of Affect in Social Cognition.

[bib6] Blascovich J., Tomaka J., Zanna M. (1996). Advances in Experimental Social Psychology.

[bib7] Borsboom D., Cramer A.O. (2013). Network analysis: an integrative approach to the structure of psychopathology. Annu. Rev. Clin. Psychol..

[bib8] Boscarino J.A. (2004). Posttraumatic stress disorder and physical illness: results from clinical and epidemiologic studies. Ann. N. Y. Acad. Sci..

[bib9] Briganti G., Decety J., Scutari M., McNally R.J., Linkowski P. (2022). Using Bayesian networks to investigate psychological constructs: the case of empathy. Psychol. Rep..

[bib10] Cañas-González B., Fernández-Nistal A., Ramírez J.M., Martínez-Fernández V. (2020). Influence of stress and depression on the immune system in patients evaluated in an anti-aging unit. Front. Psychol..

[bib11] Carlsson E., Frostell A., Ludvigsson J., Faresjo M. (2014). Psychological stress in children may alter immune response. J. Immunol..

[bib12] Cazassa M., Oliveira M., Spahr C., Shields G., Slavich G. (2020). The stress and adversity inventory for adults (adult STRAIN) in Brazilian Portuguese: initial validation and links with executive function, sleep, and mental and physical health. Front. Psychol..

[bib13] Centers for Disease Control and Prevention (2023). 2025, from Youth Risk Behavior Survey Data Summary & Trends Report.

[bib14] Charles S.T., Piazza J.R., Mogle J., Sliwinski M.J., Almeida D.M. (2013). The wear and tear of daily stressors on mental health. Psychol. Sci..

[bib15] Copeland W.E., Wolke D., Lereya S.T., Shanahan L., Worthman C., Costello E.J. (2014). Childhood bullying involvement predicts low-grade systemic inflammation into adulthood. Proc. Natl. Acad. Sci..

[bib16] Crum A.J., Jamieson J.P., Akinola M. (2020). Optimizing stress: an integrated intervention for regulating stress responses. Emotion.

[bib17] Dhabhar F.S. (2009). A hassle a day May keep the pathogens away: the fight-or-flight stress response and the augmentation of immune function. Integr. Comp. Biol..

[bib18] Dhabhar F.S. (2014). Effects of stress on immune function: the good, the bad, and the beautiful. Immunol. Res..

[bib19] Dhabhar F.S. (2019). The power of positive stress – a complementary commentary. Int. J. Biol. Stress.

[bib20] Dhabhar F.S., Malarkey W.B., Neri E., McEwen B.S. (2012). Stress-induced redistribution of immune cells—from barracks to boulevards to battlefields: a tale of three hormones. Psychoneuroendocrinology.

[bib21] Dohrenwend B.P. (2006). Inventorying stressful life events as risk factors for psychopathology: toward resolution of the problem of intracategory variability. Psychol. Bull..

[bib22] Dyson R., Renk K. (2006). Freshmen adaptation to university life: depressive symptoms, stress, and coping. J. Clin. Psychol..

[bib23] Ehlert U., Gaab J., Heinrichs M. (2001). Psychoneuroendocrinological contributions to the etiology of depression, posttraumatic stress disorder, and stress-related bodily disorders: the role of the hypothalamus-pituitary-adrenal axis. Biol. Psychol..

[bib24] Epel E.S., Crosswell A.D., Mayer S.E., Prather A.A., Slavich G.M., Puterman E., Mendes W.B. (2018). More than a feeling: a unified view of stress measurement for population science. Front. Neuroendocrinol..

[bib25] Epskamp S., Fried E.I. (2015). Package ‘bootnet’: bootstrap methods for various network estimation routines. R Package Version.

[bib26] Epskamp S., Borsboom D., Fried E.I. (2018). Estimating psychological networks and their accuracy: a tutorial paper. Behav. Res. Methods.

[bib27] Epskamp S., Costantini G., Haslbeck J., Cramer A.O., Epskamp M.S., RSVGTipsDevice S. (2017). Package ‘qgraph. https://cran.r-project.org/web/packages/qgraph/qgraph.pdf.

[bib28] Fagundes C.P., Way B. (2014). Early-life stress and adult inflammation. Curr. Dir. Psychol. Sci..

[bib29] Fletcher D., Arnold R., Arnold R., Fletcher D. (2021). Stress, well-being, and Performance in Sport.

[bib30] Foygel R., Drton M. (2010). Extended Bayesian information criteria for Gaussian graphical models. Adv. Neural Inf. Process. Syst..

[bib31] Fried E.I., Nesse R.M., Guille C., Sen S. (2015). The differential influence of life stress on individual symptoms of depression. Acta Psychiatr. Scand..

[bib32] Friedman J., Hastie T., Tibshirani R. (2008). Model selection through sparse maximum likelihood estimation for multivariate Gaussian or binary data. Biostatistics.

[bib33] Hansen K.D., Gentry J., Long L., Gentleman R., Falcon S., Hahne F., Sarkar D., Hansen M.K.D. (2015). Package ‘Rgraphviz’. https://bioconductor.statistik.tu-dortmund.de/packages/3.8/bioc/manuals/Rgraphviz/man/Rgraphviz.pdf.

[bib34] James P., Iyer A., Webb T.L. (2019). The impact of post-migration stressors on refugees' emotional distress and health: a longitudinal analysis. Eur. J. Soc. Psychol..

[bib35] Jamieson J.P., Crum A.J., Goyer J.P., Marotta M.E., Akinola M. (2018). Optimizing stress responses with reappraisal and mindset interventions: an integrated model. Anxiety Stress Coping.

[bib36] Kalin N.H. (2020). The critical relationship between anxiety and depression. Am. J. Psychiatr..

[bib37] Kessler R.C., Berglund P., Demler O., Jin R., Merikangas K.R., Walters E.E. (2005). Lifetime prevalence and age-of-onset distributions of DSM-IV disorders in the National Comorbidity Survey replication. Arch. Gen. Psychiatry.

[bib38] Keyes C.L. (2002). The mental health continuum: from languishing to flourishing in life. J. Health Soc. Behav..

[bib39] Kiecolt-Glaser J.K., Glaser R., Shuttleworth E.C., Dyer C.S., Ogrocki P., Speicher C.E. (1987). Chronic stress and immunity in family caregivers of Alzheimer's disease victims. Psychosom. Med..

[bib40] Koudela-Hamila S., Smyth J., Santangelo P., Ebner-Priemer U. (2022). Examination stress in academic students: a multimodal, real-time, real-life investigation of reported stress, social contact, blood pressure, and cortisol. J. Am. Coll. Health.

[bib41] Kroenke K., Spitzer R.L., Williams J.B. (2001). The PHQ-9: validity of a brief depression severity measure. J. Gen. Intern. Med..

[bib42] Labuschagne I., Grace C., Rendell P., Terrett G., Heinrichs M. (2019). An introductory guide to conducting the Trier social Stress Test. Neurosci. Biobehav. Rev..

[bib43] Lähdepuro A., Savolainen K., Lahti-Pulkkinen M., Eriksson J.G., Lahti J., Tuovinen S. (2019). The impact of early life stress on anxiety symptoms in late adulthood. Sci. Rep..

[bib44] Lakens D. (2022). Sample size justification. Collabra: Psychology.

[bib45] Lam J.C.W., Shields G.S., Trainor B.C., Slavich G.M., Yonelinas A.P. (2019). Greater lifetime stress exposure predicts blunted cortisol but heightened DHEA responses to acute stress. Stress Health.

[bib46] Lasselin J., Alvarez-Salas E., Grigoleit J.S. (2016). Well-being and immune response: a multi-system perspective. Curr. Opin. Pharmacol..

[bib47] Lauritzen S.L. (1996). https://books.google.com/books?hl=it&lr=&id=mGQWkx4guhAC&oi=fnd&pg=PA1.

[bib48] Mayer S.E., Prather A.A., Puterman E., Lin J., Arenander J., Coccia M., Shields G.S., Slavich G.M., Epel E.S. (2019). Cumulative lifetime stress exposure and leukocyte telomere length attrition: the unique role of stressor duration and exposure timing. Psychoneuroendocrinology.

[bib49] McElroy S., Hevey D. (2014). Relationship between adverse early experiences, stressors, psychosocial resources and wellbeing. Child Abuse Negl..

[bib50] McEwen B.S. (1998). Stress, adaptation, and disease: allostasis and allostatic load. Ann. N. Y. Acad. Sci..

[bib51] McEwen B.S., Seeman T. (1999). Protective and damaging effects of mediators of stress: elaborating and testing the concepts of allostasis and allostatic load. Ann. N. Y. Acad. Sci..

[bib52] McLoughlin E., Arnold R., Fletcher D., Spahr C.M., Slavich G.M., Moore L.J. (2022). Assessing lifetime stressor exposure in sport performers: associations with trait stress appraisals, health, well-being, and performance. Psychol. Sport Exerc..

[bib53] McLoughlin E., Arnold R., Freeman P., Turner J.E., Roberts G.A., Fletcher D., Slavich G.M., Moore L.J. (2022). Lifetime stressor exposure and psychophysiological reactivity and habituation to repeated acute social stressors. J. Sport Exerc. Psychol..

[bib54] McLoughlin E., Fletcher D., Slavich G.M., Arnold R., Moore L.J. (2021). Cumulative lifetime stress exposure, depression, anxiety, and perceived well-being in elite athletes: a mixed method study. Psychol. Sport Exerc..

[bib88] Mendes W.B., Park J. (2014). *Advances in motivation science*.

[bib55] Mission Australia (2019). Annual report 2019. https://www.missionaustralia.com.au/publications/annual-reports/annual-report-2019.

[bib56] Netea M.G., Balkwill F., Chonchol M., Cominelli F., Donath M.Y., Giamarellos-Bourboulis E.J., Golenbock D., Gresnigt M.S., Heneka M.T., Hoffman H.M., Hotchkiss R., Joosten L.A.B., Kastner D.L., Korte M., Latz E., Libby P., Mandrup-Poulsen T., Mantovani A., Mills K.H.G. (2017). A guiding map for inflammation. Nat. Immunol..

[bib57] NHS England (2023). One in five children and young people had a probable mental disorder in 2023. https://www.england.nhs.uk/2023/11/one-in-five-children-and-young-people-had-a-probable-mental-disorder-in-2023/.

[bib58] O'Donovan A., Tomiyama A.J., Lin J., Puterman E., Adler N.E., Kemeny M., Wolkowitz O.M., Blackburn E.H., Epel E.S. (2012). Stress appraisals and cellular aging: a key role for anticipatory threat in the relationship between psychological stress and telomere length. Brain Behav. Immun..

[bib59] Opsahl T., Agneessens F., Skvoretz J. (2010). Node centrality in weighted networks: generalizing degree and shortest paths. Soc. Netw..

[bib60] Pino M.C., Vagnetti R., Masedu F., Attanasio M., Tiberti S., Valenti M., Mazza M. (2020). Mapping the network of social cognition domains in children with autism spectrum disorder through graph analysis. Front. Psychiatr..

[bib61] Russell S.J., Norvig P. (2010). https://ds.amu.edu.et/xmlui/bitstream/handle/123456789/10406/artificial%20intelligence%20-%20a%20modern%20approach%20%283rd%2C%202009%29.pdf?sequence=1&isAllowed=y.

[bib62] Savage M.J., Magistro D., Hennis P.J., Donaldson J., Healy L.C., Hunter K.A., James R.M. (2024). Determining factors of physical activity and sedentary behaviour in university students during the COVID-19 pandemic: a longitudinal study. PLoS One.

[bib63] Schinke R.J., Stambulova N.B., Si G., Moore Z. (2018). International Society of sport psychology position stand: athletes' mental health, performance, and development. Int. J. Sport Exerc. Psychol..

[bib64] Scutari M., Nagarajan R. (2013). Identifying significant edges in graphical models of molecular networks. Artif. Intell. Med..

[bib65] Scutari M., Scutari M.M., MMPC H.-P. (2019). Package ‘bnlearn’: bayesian network structure learning. https://cran.r-project.org/web/packages/bnlearn/bnlearn.pdf.

[bib66] Seery M.D. (2011). Challenge or threat? Cardiovascular indexes of resilience and vulnerability to potential stress in humans. Neurosci. Biobehav. Rev..

[bib67] Seery M.D. (2013). The biopsychosocial model of challenge and threat: using the heart to measure the mind. Soc. Personal. Psychol. Compass.

[bib68] Seery M.D., Holman A.E., Silver R.C. (2010). Whatever does not kill us: cumulative lifetime adversity, vulnerability, and resilience. J. Personality Soc. Psychol..

[bib69] Shields G.S., Fassett-Carman A., Gray Z.J., Gonzalez J.E., Snyder H.R., Slavich G.M. (2023). Why is subjective stress severity a stronger predictor of health than stressor exposure? A pre-registered two-study test of two hypotheses. Stress Health.

[bib70] Shirinivas S.G., Vetrivel S., Elango N.M. (2010). Applications of graph theory in computer science: an overview. Int. J. Eng. Sci. Technol..

[bib71] Shutta K.H., De Vito R., Scholtens D.M., Balasubramanian R. (2022). Gaussian graphical models with applications to omics analyses. Stat. Med..

[bib72] Slavich G.M., Shields G.S. (2018). Assessing lifetime stress exposure using the stress and adversity inventory for adults (adult STRAIN). Psychosom. Med..

[bib73] Slavich G.M., Roos L.G., Mengelkoch S., Webb C.A., Shattuck E.C., Moriarity D.P., Alley J.C. (2023). Social safety theory: conceptual foundation, underlying mechanisms, and future directions. Health Psychol. Rev..

[bib74] Slavich G.M., Stewart J.G., Esposito E.C., Shields G.S., Auerbach R.P. (2019). The stress and adversity inventory for Adolescents (Adolescent STRAIN): associations with mental and physical health, risky behaviors, and psychiatric diagnoses in youth seeking treatment. JCPP (J. Child Psychol. Psychiatry).

[bib75] Spitzer R., Kroenke K., Williams J., Löwe B. (2006). A brief measure for assessing generalized anxiety disorder. Arch. Intern. Med..

[bib76] Stein M.B., Heimberg R.G. (2004). Well-being and life satisfaction in generalized anxiety disorder: comparison to major depressive disorder in a community sample. J. Affect. Disord..

[bib77] Syed S.A., Nemeroff C.B. (2017). Early life stress, mood, and anxiety disorders. Chronic Stress.

[bib87] Topp C.W., Østergaard S.D., Søndergaard S., Bech P. (2015). The WHO-5 Well-Being Index: a systematic review of the literature. Psychotherapy and psychosomatics.

[bib78] Turner A., Smyth N., Hall S., Torres S., Hussein M., Jayasinghe S. (2020). Psychological stress reactivity and future health and disease outcomes: a systematic review of prospective evidence. Psychoneuroendocrinology.

[bib79] Twenge J.M., Cooper A.B., Joiner T.E., Duffy M.E., Binau S.G. (2019). Age, period, and cohort trends in mood disorder indicators and suicide-related outcomes in a nationally representative dataset, 2005–2017. J. Abnorm. Psychol..

[bib80] Vagg P.R., Spielberger C.D. (1999). The job stress Survey: assessing perceived severity and frequency of occurrence of generic sources of stress in the workplace. J. Occup. Health Psychol..

[bib81] Vagnetti R., Pino M.C., Masedu F., Peretti S., Le Donne I., Rossi R., Valenti M., Mazza M. (2020). Exploring the social cognition network in young adults with autism spectrum disorder using graph analysis. Brain and Behavior.

[bib82] Varol U., Ubeda-D’Ocasar E., Cigaran-Méndez M., Arias-Buría J.L., Fernández-de-las-Peñas C., Gallego-Sendarrubias G.M., Valera-Calero J.A. (2023). Understanding the psychophysiological and sensitization mechanisms behind fibromyalgia syndrome: a network analysis approach. Pain Med..

[bib83] Vedhara K., Cox N.K., Wilcock G.K., Perks P., Hunt M., Anderson S., Lightman S.L., Shanks N.M. (1999). Chronic stress in elderly carers of dementia patients and antibody response to influenza vaccination. Lancet.

[bib84] World Health Organisation (2004).

[bib85] World Health Organization (1998).

[bib86] Wyn J. (2022). Young people's mental health. J. Appl. Youth Stud..

