# Supplementary material for: A network analysis of lifetime stressor exposure, mental health, well-being, and immune cell mobilisation to acute stressors in young adults
Source: Brain Behav Immun Health. 2026 Jan 26;52:101186. doi: 10.1016/j.bbih.2026.101186 (PMC12873736; doi:10.1016/j.bbih.2026.101186)
Supplement: Multimedia component 1 [file mmc1.docx]

**Supplementary Materials**

**Figure S1.** Schematic diagram of the procedure for the laboratory-based activity (i.e., Trier Social Stress Test [TSST]).

**Pre-TSST**

2. Participants attended a laboratory at the University of Bath and provided informed consent.

3. Participants sat resting for 20 minutes. At the end of this resting period, a finger-tip blood sample was collected.

**TSST Trial 1**

4. Participants were given instructions about the speech task.

5. Participants prepared for the speech task for 5 minutes.

6. Participants performed the speech task for 5 minutes while being video recorded.

7. Participants were given instructions about the mental arithmetic task.

8. Participants performed the mental arithmetic task for 5 minutes while being video recorded.

**Laboratory Testing Session**

**Pre-Laboratory**

1. Participants completed an online questionnaire approximately 48 hours before visiting the laboratory. This online questionnaire assessed exposure to lifetime stressors (via the Adult STRAIN), symptoms of depression (via the PHQ-9), and anxiety (via the GAD-7), and levels of well-being (via the WHO-5).

**Post-TSST**

9. Immediately after the TSST, a finger-tip blood sample was collected.

10. Participants were debriefed and thanked for their participation.
